# Extended pharmacological miosis is superfluous after glaucoma angle surgery: A retrospective study

**DOI:** 10.12688/f1000research.13756.1

**Published:** 2018-02-12

**Authors:** Hamed Esfandiari, Kiana Hassanpour, Mehdi Yaseri, Nils A. Loewen

**Affiliations:** 1 Department of Ophthalmology, School of Medicine, University of Pittsburgh, Pittsburgh, PA, USA; 2 Ophthalmic Research Center, Shahid Beheshti University of Medical Sciences, Tehran, Iran; 3 Department of Epidemiology and Biostatistics, School of Public Health, Tehran University of Medical Sciences, Tehran, Iran

**Keywords:** Trabectome surgery, ab interno trabeculectomy, pilocarpine eye drop, miotics, peripheral anterior synechiae

## Abstract

**Background: **Pilocarpine is commonly used after angle surgery for glaucoma despite a host of side effects and risks. We hypothesized that ****a pharmacological miosis during the first two months does not improve short- and long-term results of trabectome-mediated *ab interno* trabeculectomy.

**Methods:** In this retrospective comparative 1-year case series, we compared 187 trabectome surgery eyes with (P+) or without (P-) 1% pilocarpine for two months. Primary outcome measures were the surgical success defined as intraocular pressure (IOP) ≤ 21 mmHg and decreased ≥ 20%, and no secondary glaucoma surgery. Secondary outcome measures were the number of glaucoma medications, complications, and IOP.

**Results: **We categorized 86 (46%) eyes as P- and 101 (54%) eyes as P+. The mean age was 69.8±10.1 in P- and 70.5±9.4 in P+ (P=0.617) with equal gender distribution (P=0.38). The cumulative probability of qualified success at 12 months was 78.1% in the P- and 81% in the P+ (P=0.35). The IOP was decreased significantly from 20.2±6.8 mmHg at baseline to 15.0±4.8 mmHg at 12 months follow-up in P- (P=0.001) and 18.8±5.3 and 14.7±4.0, respectively (P=0.001). The medications decreased significantly from 1.4±1.2 in P- and 1.4±1.2 in P+ at baseline to 1.0±1.2 ****and 0.7±1.0, respectively (P=0.183). P- and P+ did not differ in IOP or medications (all P>0.05). In Multivariate Cox Regression models, the baseline IOP and central corneal thickness were associated with failure.

**Conclusions:** ****Use of postoperative pilocarpine does not improve the efficacy of trabectome surgery.

## Introduction

*Ab interno* angle surgeries that disrupt or bypass the trabecular meshwork (TM) are bleb-less procedures without the need for office procedures and application of antifibrotics, the frequency of postoperative visits can be reduced ^1^. Most surgeons administer pilocarpine hydrochloride 1% for two months to decrease the apposition between iris root and trabecular meshwork and minimize the risk of peripheral anterior synechiae (PAS) formation. Although this seems intuitive, miotics shallow the anterior chamber when the ciliary muscle contracts and can cause angle closure in phakic eyes ^2^. There is no correlation between the degree of PAS formation and the amount of intraocular pressure (IOP) elevation after trabectome surgery ^3, 4^, a common and mature form of *ab interno* trabeculectomy ^5^ appropriate for different types of glaucomas, including angle closure ^6^, pigmentary ^7^, exfoliative ^8^, steroid-induced ^9^, uveitic ^10^, and advanced glaucomas ^11^, as well as failed trabeculectomy and glaucoma shunt procedure ^12, 13^.

Pilocarpine is a nonselective muscarinic receptor agonist that has been used extensively in the medical management of glaucoma for almost 150 years ^14^. It attaches to muscarinic receptors on ciliary smooth muscle and causes contraction of the longitudinal fibres. Following longitudinal muscle contraction, scleral spur is pulled back leading to expansion of juxtacanalicular portion of the TM and Schlemn’s canal (SC) and consequently enhance the aqueous outflow ^15, 16^. However, a TM-mediated IOP effect, for instance by adding cataract surgery, becomes negligible after trabectome surgery ^17, 18^, because the aqueous can bypass the remaining TM. Patients dislike miotics because of their side effects of headaches, nearsightedness, dim vision and more serious problems that include retinal detachment, worsening intraocular inflammation, paradoxical increase in IOP and cataract formation ^19– 23^.

Since there is no evidence that pilocarpine decreases PAS formation after trabectome surgery ^24^, along with the fact that PAS is not a risk factor for trabectome failure established without doubt ^25^, we hypothesized that postoperative pilocarpine does not improve the outcome and stopped its use in our practice. The present study compares the outcome of operation with pilocarpine versus no pilocarpine to test out hypothesis.

## Methods

Institutional Review Board approval was obtained from the University of Pittsburgh Human Subjects Research Committee (approval number: PRO13120466). An informed consent from the patients was waived due to the low risk nature of this retrospective study. We followed the tenets of the Declaration of Helsinki and regulations of the Health Insurance Portability and Accountability Act.

### Study design

This retrospective comparative study included all patients who underwent Trabectome surgery between July 2012 and October 2017 at the Eye and Ear Institute of the University of Pittsburgh, School of Medicine, in Pittsburgh, PA, United States. Patients were identified using current procedural terminology codes of Eye and Ear Institute, University of Pittsburgh.

### Participants

Patients were included regardless of same-session phacoemulsification, because the impact of same-session phacoemulsification on IOP is negligible in trabectome surgery ^17, 18^. Eyes were categorized as P- if no pilocarpine was used after the surgery, and P+ if pilocarpine was administered postoperatively.

Exclusion criteria were a history of incisional and angle surgeries, combined glaucoma surgeries, use of pilocarpine in the contralateral eye, any systemic medication with parasympathomimetic activities, and less than 3 months follow-up.

Information collected included demographic data, types of glaucoma, pre- and postoperative intraocular pressures (IOP), baseline ocular biometric characteristics including axial length (AL), central corneal thickness (CCT), anterior chamber depth (ACD), number of pre-and postoperative glaucoma medications, visual field mean deviation (MD), visual field index (VFI), type of surgery, use of pilocarpine eye drop after the surgery, and intra- and postoperative complications.

The primary outcome measure was success and defined as 5 < IOP ≤ 21 mmHg, ≥ 20% reduction of IOP from baseline at two consecutive visits, no need for further glaucoma surgery, and no loss of light perception. Qualified success was defined as achieving success with or without medications. Patients who achieved success without medication were labeled as complete success. A Kaplan Meier survival analysis was used to evaluate success rates. The secondary outcome measures were IOP, glaucoma medications, and complications.

### Surgical technique

In the case of combined phacoemulsification, trabectome (NeoMedix, Tustin, CA, USA) portion was performed first. Both the patient’s head and microscope were tilted 30° away from the surgeon. A temporal 1.6 mm incision was made 2 mm anterior to the limbus and parallel to the iris plane. The trabectome was inserted through the incision and positioned across the AC along the nasal angle. The tip of the trabectome was inserted into Schlemm’s canal and engaged with the TM. The aspiration and ablation was activated with the power set to 0.8 to 1W and the TM removed over approximately 160°. The tip was withdrawn from anterior chamber and the incision was hydrated to achieve watertightness. All glaucoma medications were discontinued after the surgery and were resumed if IOP was not within target range. In P+, 1% pilocarpine was used 4 times a day for one month followed by three times a day for a second month.

### Statistical analysis

All analyses were performed using SPSS software (SPSS Statistics for Windows, Version 25, Armonk, NY, IBM Corporation). Frequency, percent, mean±SD, median, and range were used to describe the data. To evaluate the baseline differences between the two study groups, we used the T-test, Mann Whitney, and Chi-Square test. To compare the change in IOP and number of medications between the two groups or within a group, we used an interaction analysis within a linear mixed model. To compare the amount of IOP reduction between the groups adjusted for the baseline values, we used an Analysis of Covariance (ANCOVA). Kaplan-Meier survival plots were constructed to assess the long-term survival rates and compared by the log-rank test. A Cox proportional Hazard model was used to find risk factors for failure and to estimate the adjusted Hazard ratio of each factor. In the last step to obtain the most important factors, we used a backward variable elimination method based on the LR test. Statistical significance was set at p< 0.05. Success was defined as IOP <21 mmHg and a >20% reduction from baseline with no need for additional glaucoma surgery.

## Results

### Baseline characteristics

A total of 187 eyes were included in this study. Eighty-six (46%) eyes in P- did not receive pilocarpine, while 101 (54%) in P+ did ( Table 1). All patients were phakic at the time of surgery. Phacoemulsification was combined in 147 (79%) eyes (TP) while 40 eyes had trabectome surgery alone (T). The mean age of participants was 69.8±10.1 in P- and 70.5±9.4 in P+. Primary open-angle glaucoma was the most common diagnosis in both groups (73.3% and 63.2% in P- and P+, respectively, P=0.64). There were no significant differences between the study groups in terms of gender, preoperative intraocular pressure, central corneal thickness, axial length, visual field mean deviation, anterior chamber depth, baseline number of glaucoma medications, and type of glaucoma ( Table 1).

**Table 1.** Baseline clinical characteristics of patients in P- and P+.

		All	P-	P+	p-value
**Age**		70.1±9.7	69.8±10.1	70.5±9.4	0.617 †
**Gender**	***Female***	99 (52.9%)	44 (52.1%)	55 (54.5%)	0.380 *
	***Male***	88 (47.1%)	42 (48.8%)	46 (45.5%)	
**Glaucoma type**	***POAG***	68.4%	73.3%	63.2%	0.640 *
	***CACG***	11.1%	8.3%	14.0%	
	***XFG***	10.3%	10.0%	10.5%	
	***Others***	10.3%	8.3%	12.3%	
**Axial length (mm)**		24±2.2	24±2.9	24.1±1.2	0.915 †
**CCT**		544±40	549±44	540±37	0.154 †
**AC depth (mm)**		3.1±0.6	3.05±0.6	3.2±0.6	0.061 †
**Lens thickness (mm)**		4.4±0.8	4.5±0.6	4.3±1.0	0.129 †
**HVF MD**		-6.6±7.9	-7.6±8.7	-5.9±7.2	0.079 ‡
**IOP (mmHg)**		19.5±6.1	20.2±6.8	18.8±5.3	0.120 †
**Medications**		1.4±1.2	1.4±1.2	1.4±1.2	0.902 ‡

†Based on T-test. *Based on Chi-square. ‡ Based on Mann-Whitney test. AC: Anterior chamber, CACG: Chronic angle closure glaucoma, CCT: Central corneal thickness, HVF MD: Humphrey Visual Field Mean Deviation, IOP: intraocular pressure, POAG: primary open angle glaucoma, XFG: exfoliation glaucoma.

### Surgical success and risk factors for failure

**Figure 1.**
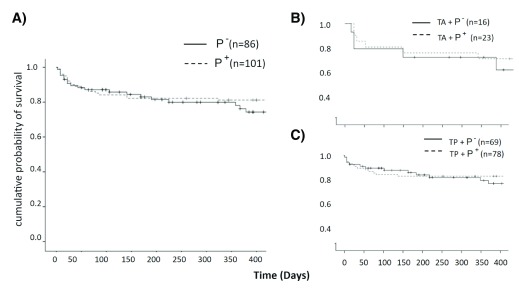
Kaplan-Meier survival plots for P- and P+ with success defined as a final intraocular pressure of ≤ 21 mmHg and a 20% reduction from baseline. Success rates were similar in both groups. Survival plots of P- and P+ for subgroup analysis separated by **B**) glaucoma surgery alone and **C**) same session phacoemulsification.

Kaplan-Meier survival curves ( Figure 1) indicated a mean survival of 34.03±2.35 months in P- and 38.32±1.94 months in P+ with no statistically significant difference between two groups (log rank:0.87 P=0.35). The addition of phacoemulsification did not make a difference on survival (T: 33.9±3.2 months; TP: 32.3±1.7, log-rank:0.81 P=0.36). Similarly, pilocarpine did not have a significant effect on survival in T or TP: T in P- had a survival of 32.5±5.4 and T in P+ had a survival of 33.3±3.9 months (log-rank:0.06, P=0.8). TP in P- had a survival of 33.6±2.4 and TP in P+ had a survival of 36.2±2.2 months (log-rank:0.81, P=0.36). The multivariate Cox regression model was stratified by group, age, baseline IOP, number of medications, glaucoma type, CCT, AC depth, lens thickness, axial length, Humphrey visual field (HVF) MD and VFI ( Table 2). The final model with backward elimination only included baseline IOP (mmHg) and CCT (micron) with HR of 4.1 for 22<IOP<28, 3.7 for IOP>28, and 1.03 for CCT. This means that in patients with 22<IOP<28 the risk of failure was 4.1 times higher compared to patients with IOP<18 (P=0.015). Every 10 micron increase in baseline CCT increased the risk of failure by 30% (P=0.005).

**Table 2.** Hazard ratios of risk factors for failures. Results from multivariate cox proportional hazard regression model. IOP, intraocular pressure; T, trabectome surgery; TP, trabectome surgery with phacoemulsification; CCT, central corneal thickness; AC, anterior chamber depth; HVF MD, Humphrey Visual Field Mean Deviation; HVF VFI, Humphrey Visual Field visual field index.

Risk factor	HR (95% CI)	p-value
Pilocarpine use	0.62 (0.14-2.63)	0.52
Age	0.99 (0.91-1.09)	0.45
Gender	0.57 (0.16-2.0)	0.38
Glaucoma type	0.94 (0.09-12.3)	0.97
18<IOP<22	1.36 (0.33-5.5)	0.66
22<IOP<28	4.1 (1.31-12.84)	0.015
IOP>28	3.74 (1.13-12.35)	0.03
Baseline medications	2.11 (0.92-4.83)	0.07
T vs TP	1.61 (0.37-6.9)	0.52
CCT	1.03 (1.01-1.04)	0.005
AC depth	0.84 (0.23-3.03)	0.79
Axial length	1.18 (0.66-2.14)	0.56
Lens thickness	0.5 (0.18-1.37)	0.18
HVF MD	1.0 (0.99-1.02)	0.46
HVF VFI	1.008 (0.98-1.02)	0.44

### IOP change

**Figure 2.**
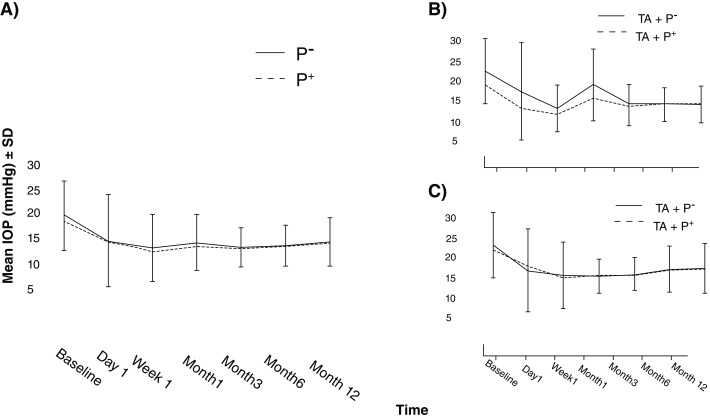
Intraocular pressure (IOP) in P- and P+ groups. **A**) IOP in P- was similar to P+ through follow-up duration. IOP in both groups for subgroup analysis separated by **B**) glaucoma surgery alone. Patients who received pilocarpine showed lower IOPs during the first month but the difference was not statistically significant. **C**) Same session phacoemulsification. Error bars represent standard deviations.

IOP was decreased significantly from 20.2±6.8 mmHg at baseline to 15.0±4.8 mmHg at 12 months follow-up in P- (P=0.001, Figure 2). The corresponding numbers for P+ were 18.8±5.3 and 14.7±4.1, respectively (P=0.001). There was no significant difference in IOP at each follow-up visit between the two groups ( Table 3). In subgroup analysis, considering the effect of phacoemulsification, there was no significant difference between IOPs of both groups in T and TP throughout the course of follow-up ( Figure 2). Seven (8.1%) cases in P- and five (5%) in P+ experienced IOP spikes. There was no significant difference between the groups (P=0.153, based on the Chi-square test). The baseline number of glaucoma medications was 1.4±1.2 in P- and 1.4±1.2 in P+ (P=0.910). At month 12, the number of glaucoma medications was 1.0±1.2 drops in P- and 0.7±1.0 in P+ (P=0.082, Table 4).

**Table 3.** Mean intraocular pressure (IOP) and IOP change over time.

	P-	P+	p-value
Baseline	20.2±6.8	18.8±5.3	0.12
Day 1	15.1±9.0	15.0±7.0	0.482
Week 1	13.7±6.7	13.0±4.0	0.427
Month 1	14.9±5.4	14.0±5.1	0.814
Months 3	13.9±3.7	13.6±3.7	0.417
Month 6	14.3±4.0	13.9±3.6	0.301
Month 12	15.0±4.8	14.7±4.0	0.274

**Table 4.** Mean number of glaucoma medications.

	P-	P+	p-value
Baseline	1.4±1.2	1.4±1.2	0.91
Month 1	0.5±1.0	0.3±0.7	0.413
Months 3	0.4±1.0	0.3±0.7	0.523
Month 6	0.5±1.0	0.4±0.8	0.569
Month 12	1.0±1.2	0.7±1.0	0.183

The postoperative hyphema was observed in 49% of the eyes. The mean duration of hyphema was 10.68±9.96 days in P- and 12±4.30 days in P+ (P= 0.48).

Raw data collected from all study participants. Coding scheme for the data contained in Word document Click here for additional data file. Copyright: © 2018 Esfandiari H et al. 2018 Data associated with the article are available under the terms of the Creative Commons Zero "No rights reserved" data waiver (CC0 1.0 Public domain dedication).

## Discussion

Miotics are commonly used at the conclusion of cataract surgery to retract a floppy iris from the incision ^26^ or to secure a sulcus lens implant after a complex cataract surgery ^27^. It is, therefore, not surprising that *ab interno* glaucoma surgeons resort to the same medications despite reports of worsening of iritis ^28, 29^, retinal detachment and further angle narrowing, as the most serious complications ^19– 23^. Interestingly, a narrow angle is not a contraindication to trabectome surgery and yields similar results to wide angles ^6^. In the current study, the survival, IOP reduction, and duration of postoperative hyphema were comparable regardless of pilocarpine use. Both groups experienced an IOP reduction like that observed in previous studies ^5, 6, 30^.

It is unclear what may cause a late failure after trabectome surgery as the aqueous humor is not redirected into a conjunctival bleb maintained by antifibrotics or into tube shunts and microbypasses that cause a chronic foreign body reaction. Certainly, descemetization ^31^ of the outer wall of Schlemm’s canal and PAS formation may be causes of surgical failure ^4, 5, 32^, but it is not established whether miotics prevent this. Additionally, the incidence of PAS ranges from 20% to 40% which does not match the relatively high success rate observed with this procedure ^3– 5^. In one study by Minckler *et al*., the incidence of PAS was 38% ^33^, while in another study by Wang *et al*. without pilocarpine, the incidence of PAS was 20% ^4^.

The results of our study match established findings from other studies that higher baseline IOP is associated with larger IOP reduction ^5, 25^. It is consistent with the fact that after removing the site of the highest outflow resistance by trabectome, aqueous outflow would be limited only by downstream pathway. While post-trabectome IOPs tend to be similar in all patients, those with higher preoperative IOP experience larger IOP reduction ^11^.

Our study indicates a link between corneal biomechanics and risk of failure following trabectome surgery. Corneal thickness serves as an *in vivo* surrogate marker of corneal rigidity and is associated with biomechanical properties of the cornea ^34^ and likely the sclera ^35– 37^. After trabecular ablation, outflow is only limited by the remaining outflow resistance in collector channels and episcleral veins. The collector channels and the intrascleral plexus have features that indicate they can regulate the outflow resistance like the remainder of the vascular system ^38, 39^. This novel finding invites further researches to clarify the role of corneal and scleral biomechanics in predicting the behavior of the downstream pathway in angle surgery.

This study was limited by its retrospective nature and number of patients. It was powered to detect a difference of 2 mmHg between the two groups (power 0.8, alpha 0.5), a clinically meaningful difference. It would have required close to 360 individuals in each group to detect a 1 mmHg difference. Patients were not randomized or matched and PAS was not assessed systematically.

In conclusion, our study indicates that administering pilocarpine eye drop after trabectome surgery does not improve the outcome of the procedure. Given the multitude of side effects, we recommend it be avoided. A thicker cornea was associated with a higher risk of failure.

## Data availability

The data referenced by this article are under copyright with the following copyright statement: Copyright: © 2018 Esfandiari H et al.

Data associated with the article are available under the terms of the Creative Commons Zero "No rights reserved" data waiver (CC0 1.0 Public domain dedication). 

Dataset 1: Raw data collected from all study participants. Coding scheme for the data contained in Word document. DOI, http://dx.doi.org/10.5256/f1000research.13756.d193749 ^40^
