# Supplementary material for: Extended pharmacological miosis is superfluous after glaucoma angle surgery: A retrospective study
Source: F1000Res. 2018 Feb 12;7:178. [Version 1] doi: 10.12688/f1000research.13756.1 (PMC5897784; doi:10.12688/f1000research.13756.1)
Supplement: Raw data collected from all study participants. Coding scheme for the data contained in Word document [file f1000research-7-14948-s0000.tgz › d7cc8616-9268-450c-abbd-1d8a05e97890_Dataset_1.zip!/Dataset 1/Final Comprehensive Trabectome Database Codebook (1).docx]

Default binary: Y = 1, N = 2

Missing data code: 999

Dates format: 06/20/1999

GENDER:	1: Female

2: Male

EYE:

1: Right

2: Left

LENS STATUS

1: phakic

2. pseudophakic

3. aphakic

AL = axial length

(two digits, no units necessary)

CCT = Central cornea thickness

(three digits, no units necessary)

GLACUOMA TYPE:

1: POAG

2: Chronic Narrow Angle Glaucoma

3: Pseudoexfoliation

4: Pigment Dispersion Glaucoma

5: Uveitic/Inflammatory

6: Mixed Mechanism

7: Other Open Angle Glaucoma

8: Other Closed Angle Glaucoma

9: unspecified

10: Congenital Glaucoma

11: Juvenile Glaucoma

12: Congenital Ectropion Uveae

13: Sturge Weber

14: other congenital

15: Secondary to Lens

16: Trauma

17: steroid induced

GLAUCOMA [AGS](http://www.aao.org/assets/68cdbc7d-35ba-47ce-b95e-726eb503fe3e/635577092075270000/how-to-code-for-different-stages-of-glaucoma-using-icd-10-codes-pdf?inline=1) STAGE:

1: Mild

2: Moderate

3: Severe

4: Indeterminate/unspecified

HVF MD (3 digits, no units necessary)

HVF VFI (2 digits, no units necessary)

BVA = best visual acuity w/ Snellen VA

(pinhole, manifest refraction, etc)

If worse, enter CF, HM, LP, NLP, ENUC

Baseline meds

1 digit, no units;

- Combo meds count as 2

- PO med (Diamox) counts as 1

count

Prior Trab:

1: yes

2: no

Prior Tube:

1: yes

2: no

Prior Trabectome:

1: yes

2: no

Prior LPI:

1: Yes

2: No

Prior ALT/SLT:

1: Yes

2: No

Prior CataractSx:

1: Yes

2: No

Complicated Cat Sx

1: Yes

2: No

Pre Retina:

1: Yes

2: No

Pre Vitrectomy:

1: Yes

2: No

Combined Surgery (same for all yes/no questions)

1: yes

2: no

Combined with cataract surgery:

1: yes

2: no

Combined with glaucoma surgery:

1: yes

2: no

POSTOP VISITS

Post-op IOP: 2 digits, no units; please use most relevant measurement

Post-op Meds: 1 digit, no units;

- Combo meds count as 2

- PO med (Diamox) counts as 1

- Pilocarpine does NOT count

Pilocarpine:

1: yes

2: no

Post-op Hyphema:

1: yes

2: no

Need for further surgery:

1: yes

2: no

If need for further surgery, enter under comments tab using code types below or explain otherwise if type not listed

Type of additional surgery

1: AC Wash

2: Repeat GATT

3: YAG goniopuncture

4: Bleb revision with conjunctivoplasty (for bleb leak)

5: Iridovitrectomy (for aqueous misdirection)

6: IOL removal

7: Sutured IOL
