# Supplementary material for: Extended pharmacological miosis is superfluous after glaucoma angle surgery: A retrospective study
Source: F1000Res. 2018 Feb 12;7:178. [Version 1] doi: 10.12688/f1000research.13756.1 (PMC5897784; doi:10.12688/f1000research.13756.1)
Supplement: Peer review history

# Peer review: Referee response for version 1

**DOI:** 10.5256/f1000research.14948.r33024

**Published:** 2018-04-11

**Authors:** Sylvain Roy

**Affiliations:** 1 Glaucoma Research Center, Montchoisi Clinic, Swiss Vision Network, Lausanne, Switzerland

This is a well written article about surgical technique and pharmacological requirements after glaucoma angle surgery. The details provided in this study will help better understanding this new technology and simplify postoperative management, while eliminating the burden of an old-fashioned miotic medication, i.e. the pilocarpine.

 I have few minor comments pertaining to this manuscript.

- Page 4 §1: For the sake of precision I would propose the following: Success was defined as IOP <21 mmHg and a >20% reduction from baseline with no need for additional glaucoma surgery and no loss of light perception.
- Page 4 Table 1: Please provide the units for Age (years); CCT (µm)
- Page 6 Figure 2 B and 2 C: For the sake of precision I would recommend also indicating the legend on the Y-axis, e.g. Mean IOP (mmHg) ± SD for Figure 2 B and Figure 2 C.

I have read this submission. I believe that I have an appropriate level of expertise to confirm that it is of an acceptable scientific standard.

# Peer review: Referee response for version 1

**DOI:** 10.5256/f1000research.14948.r32413

**Published:** 2018-03-28

**Authors:** Don S. Minckler

**Affiliations:** 1 Gavin Herbert Eye Institute, University of California, Irvine, CA, USA

This paper by Estandian et al. will be greatly appreciated by Trabectome and other glaucoma angle-surgeons. The logic of the discussion is outstanding and the references inclusive. I was an early enthusiast of Trabectome and well-remember that the rationale for utilizing pilocarpine post-operatively was based on non-proven concepts and the then fuzzier understanding of angle physiology. Not utilizing pilocarpine after these surgeries will greatly simplify the whole follow-up with great benefit to patients.  

 I have no significant revisions to suggest.

I have read this submission. I believe that I have an appropriate level of expertise to confirm that it is of an acceptable scientific standard.
